# Immune checkpoint inhibitor–related pancreatitis with fulminant type 1 diabetes mellitus: a case report

**DOI:** 10.3389/fimmu.2026.1906731

**Published:** 2026-09-15

**Authors:** Li Niu, Shiqi Sun, Lingyue Zhang, Yanyan Song, Qi Huang, Jiazhong Sun

**Affiliations:** 1 Department of Endocrinology, Key Laboratory for Molecular Diagnosis of Hubei Province, The Central Hospital of Wuhan, Tongji Medical College, Huazhong University of Science and Technology, Wuhan, China; 2 Department of Laboratory Medicine, The Sixth Hospital of Wuhan, Affiliated Hospital of Jianghan University, Wuhan, China; 3 Department of Endocrinology, Zhongnan Hospital of Wuhan University, Wuhan, China

**Keywords:** acute pancreatitis, diabetic ketoacidosis, fulminant type 1 diabetes mellitus, immune checkpoint inhibitors, immune-related adverse events, toripalimab

## Abstract

**Background:**

Immune checkpoint inhibitors (ICIs) are widely used in tumor therapy, and the immune-related adverse events (irAEs) they induce can involve multiple organs. As an organ with both endocrine and exocrine functions, pancreatic involvement may manifest as acute pancreatitis, new-onset diabetes mellitus, or even a combination of both. Among these, ICI-induced acute pancreatitis concurrent with fulminant type 1 diabetes mellitus (FT1DM) is extremely rare but highly critical and easily missed in clinical practice.

**Case presentation:**

A 59-year-old male patient with squamous cell carcinoma of the left base of the tongue received toripalimab combined with chemotherapy. On day 7 after treatment, he presented with nausea, vomiting, and altered mental status. Emergency examination revealed hyperglycemia (17.4 mmol/L), severe diabetic ketoacidosis (blood ketones 9.52 mmol/L, pH 7.28), and significantly elevated serum amylase (370 U/L). Abdominal CT showed swelling of the body and tail of the pancreas with surrounding exudation, consistent with acute pancreatitis. His glycated hemoglobin (HbA1c) was normal (5.4%), pancreatic autoantibodies were negative, and islet function was completely lost. He was diagnosed with toripalimab-associated acute pancreatitis (Grade 3) concurrent with FT1DM. ICIs were permanently discontinued immediately. The patient was treated with fasting, fluid replacement, somatostatin to inhibit pancreatic enzyme secretion, and intensive insulin pump therapy for glycemic control. Glucocorticoids were not administered. After 11 days of comprehensive treatment, his symptoms resolved, and he was discharged upon normalization of pancreatic enzymes and blood glucose levels.

**Conclusion:**

PD-1 inhibitors can simultaneously impair both the endocrine and exocrine functions of the pancreas, leading to the coexistence of acute pancreatitis and FT1DM. Clinicians must maintain a high index of suspicion for this dual injury. For patients receiving ICI therapy, emergency evaluation of pancreatic function is warranted once gastrointestinal symptoms or unexplained hyperglycemia/ketosis occur. Early drug withdrawal and individualized management centered on supportive care can effectively prevent life-threatening complications. Notably, this case represents the first report of such dual pancreatic injury in a patient with locally advanced tongue base squamous cell carcinoma receiving first-cycle toripalimab combined with paclitaxel plus cisplatin chemoimmunotherapy, with distinct atypical clinical manifestations and a faster steroid-free recovery trajectory compared with the single previously published toripalimab-associated case.

## 1 Introduction

In recent years, immune checkpoint inhibitors (ICIs), represented by programmed cell death protein 1 (PD-1) and its ligand (PD-L1) inhibitors, have achieved breakthrough progress in the treatment of various solid tumors, significantly prolonging patient survival (1). However, alongside their widespread application, the occurrence of immune-related adverse events (irAEs) has increasingly drawn attention. irAEs can involve multiple systems, including the skin, gastrointestinal tract, liver, lungs, and endocrine glands. Their pathogenesis is primarily attributed to “off-target” attacks on normal tissues following T-cell activation (2).

The pancreas is a unique organ that possesses both exocrine (secreting digestive enzymes) and endocrine (secreting hormones such as insulin) functions. Consequently, when the pancreas becomes a target of irAEs, the clinical manifestations can be more complex. The spectrum of ICI-related pancreatic injury is broad, encompassing asymptomatic elevations in amylase/lipase, symptomatic acute pancreatitis (AP), new-onset diabetes mellitus, and even chronic pancreatitis or pancreatic exocrine insufficiency (3). Although the overall incidence is low (approximately 0.3%–1.4% for symptomatic pancreatitis), its insidious onset and rapid progression can lead to multiple organ dysfunction syndrome or even death if not promptly recognized and managed (4).

Of particular concern is the potential for ICIs to simultaneously impair both the endocrine and exocrine tissues of the pancreas, resulting in the coexistence of acute pancreatitis and diabetes mellitus. Among these, fulminant type 1 diabetes mellitus (FT1DM) is a distinct and critical subtype of diabetes, first systematically described by Japanese scholar Imagawa et al. in 2002 (5). Its core characteristics include an extremely abrupt onset (often within 24 hours), complete and irreversible loss of islet β-cell function, and hyperglycemia accompanied by severe diabetic ketoacidosis. Meanwhile, glycated hemoglobin (HbA1c), which reflects historical glycemic control, is only mildly elevated (typically <8.7%), indicating that the hyperglycemia occurred recently (5). Although reports of ICI-induced FT1DM have been increasing year by year, cases concurrent with acute pancreatitis remain exceedingly rare.

This article reports a case of a patient with squamous cell carcinoma of the base of the tongue treated with the PD-1 inhibitor toripalimab, who developed acute pancreatitis and FT1DM concurrently shortly after drug administration. Combined with a review of the current literature, we discuss the clinical features, potential pathogenesis, and individualized treatment strategies of this condition, aiming to enhance clinicians’ awareness of this rare but fatal complication. Importantly, this case carries unique novel clinical observations that differ substantially from the only prior published toripalimab-related concurrent pancreatitis-FT1DM case, including ultra-early toxicity onset after first-cycle chemoimmunotherapy, atypical non-abdominal-dominant symptoms, and accelerated recovery under steroid-free supportive management.

## 2 Case presentation

### 2.1 Clinical information

A 59-year-old male patient presented to the Department of Head and Neck Surgery at our hospital in January 2026, complaining of “a mass at the left base of the tongue accompanied by left cervical lymphadenopathy for one month.” Electronic laryngoscopy and biopsy pathology confirmed a diagnosis of moderately-to-poorly differentiated squamous cell carcinoma of the left base of the tongue (cT2N2M0, Stage IVA). Past medical history was significant for hypertension for three years, well-controlled with regular oral telmisartan 80 mg once daily. He denied any history of diabetes mellitus, thyroid disease, systemic autoimmune diseases, or chronic alcohol consumption. Family history was unremarkable.

On January 12, 2026, the patient received his first cycle of antitumor therapy: toripalimab 240 mg intravenously on day 1, combined with TP regimen chemotherapy (paclitaxel plus cisplatin). The treatment was well-tolerated without infusion-related reactions.

### 2.2 Disease progression and admission

Five days after treatment (January 17), the patient began to experience dizziness, fatigue, and significantly decreased appetite. In the early morning of day 7 (January 19), he developed sudden nausea and vomiting of yellowish-white gastric contents 4–5 times, accompanied by dry mouth and polydipsia. His family noticed lethargy and delayed responsiveness, prompting an immediate visit to a local hospital. Emergency evaluation revealed a markedly elevated random blood glucose level of 17.4 mmol/L, strongly positive urine ketones (+++), and arterial blood gas analysis indicating metabolic acidosis. He was subsequently transferred emergently to the Intensive Care Unit of our hospital.

Upon admission, physical examination showed: temperature 36.9°C, heart rate 102 beats/min, respiratory rate 24 breaths/min, and blood pressure 142/88 mmHg. The patient exhibited apathy and dry skin and mucous membranes. Bilateral lung sounds were clear, and cardiac rhythm was regular. The abdomen was flat and soft, with mild tenderness in the upper abdomen, but no rebound tenderness or muscular guarding was noted. Bowel sounds were normal.

### 2.3 Auxiliary examinations and diagnosis

Laboratory test results were as follows:

Metabolism and Endocrinology: Random blood glucose 17.40 mmol/L; blood ketones 9.52 mmol/L; arterial pH 7.28; HCO_3_^-^ 14.2 mmol/L; lactate 3.50 mmol/L. Glycated hemoglobin (HbA1c) was 5.4% (normal range: 4.0%–6.0%). Fasting C-peptide was <0.05 ng/mL, indicating complete islet failure.

Pancreatic Enzymes: Serum amylase 370 U/L (reference value <100 U/L); lipase 68 U/L (reference value <60 U/L).

Immunology: Glutamic acid decarboxylase antibody (GAD-Ab), islet cell antibody (ICA), and insulinoma-associated antigen-2 antibody (IA-2 Ab) were all negative.

Imaging: Non-contrast computed tomography (CT) of the upper abdomen performed on January 19, 2026 (**Figure 1**) revealed diffuse enlargement of the body and tail of the pancreas with ill-defined margins. Patchy exudative shadows were observed in the peripancreatic fat spaces. No pancreatic necrosis or pseudocyst formation was seen, consistent with acute interstitial edematous pancreatitis. We further excluded common non-immune triggers for acute pancreatitis through clinical and laboratory screening. No gallstones, biliary dilatation or gallbladder wall thickening were detected on the unenhanced abdominal CT, and liver function tests including total bilirubin and alkaline phosphatase remained within normal limits, which ruled out gallstone-induced pancreatitis. Admission fasting lipid panel showed normal triglyceride levels, eliminating hypertriglyceridemia as a precipitating factor. The patient also denied a history of chronic heavy alcohol intake, so alcoholic pancreatitis was not considered.

**Figure 1:**
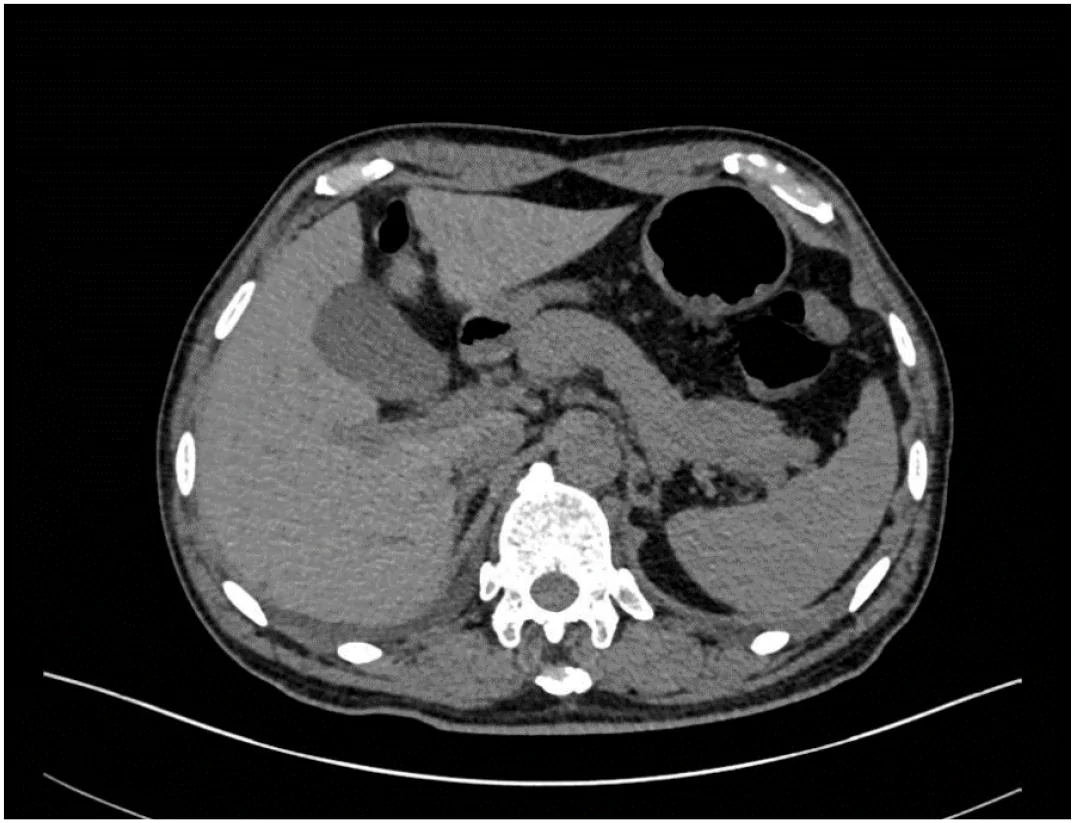
Abdominal non-contrast CT scan of the patient. Obvious swelling of the pancreatic body and tail and inflammatory exudation around the pancreas are seen, consistent with immune checkpoint inhibitor-related acute pancreatitis.

Based on the above findings, the patient developed typical FT1DM (abrupt onset, ketoacidosis, normal HbA1c, negative islet autoantibodies, and absent C-peptide) and acute pancreatitis (abdominal pain, serum amylase >3 times the upper limit of normal [ULN], and CT confirmation) on day 7 post-toripalimab administration. Common etiologies such as cholelithiasis, alcohol abuse, and hypertriglyceridemia were excluded. According to the CTCAE (Common Terminology Criteria for Adverse Events) version 5.0 criteria, the pancreatitis was graded as Grade 3 (requiring hospitalization). The final diagnoses were:

1. Toripalimab-associated acute pancreatitis (Grade 3);
2. Toripalimab-associated fulminant type 1 diabetes mellitus;
3. Squamous cell carcinoma of the left base of the tongue (cT2N2M0, Stage IVA);
4. Essential hypertension, Grade 2 (moderate risk).

### 2.4 Treatment course

In accordance with the NCCN (National Comprehensive Cancer Network) guidelines for the management of immunotherapy-related toxicities (6), toripalimab and subsequent chemotherapy were permanently discontinued immediately, given the patient’s Grade 3 pancreatitis and Grade 4 hyperglycemia (life-threatening).

The treatment regimen was as follows:

Pancreatitis Management: Strict fasting and nil per os (NPO) status were maintained, along with continuous gastrointestinal decompression. Intravenous fluid replacement was administered to maintain water and electrolyte balance. Somatostatin was continuously infused intravenously (3000 μg/24h) to inhibit pancreatic enzyme secretion. Pantoprazole was given intravenously for acid suppression, and piperacillin-tazobactam was used prophylactically for infection prevention.

Glycemic Management: Continuous subcutaneous insulin infusion (CSII) via an insulin pump was initiated immediately, with an initial basal rate of 0.1 U/kg/h. Mealtime bolus doses were dynamically adjusted based on hourly blood glucose monitoring. The therapeutic goals were to resolve ketosis within 24 hours and stabilize blood glucose levels within 72 hours.

Supportive Care: Vital signs, fluid intake and output, electrolytes (especially potassium and sodium), serum amylase/lipase, and blood ketones were closely monitored.

On day 2 of treatment, the patient’s nausea and vomiting resolved, and his mental status improved. By day 3, blood ketones turned negative, and arterial pH returned to normal. On day 5, serum amylase decreased to 128 U/L. On day 11, the patient was in good general condition and was transitioned to a multiple daily injection regimen consisting of bedtime glargine insulin combined with pre-meal aspartate insulin (three times daily). His blood glucose was well-controlled (fasting: 6–8 mmol/L; postprandial: 8–10 mmol/L), and he was subsequently discharged. Regular follow-up visits were scheduled at the outpatient clinics of both the Endocrinology and Oncology departments.

The full chronological sequence of immunotherapy administration, symptom onset, auxiliary examinations, intervention initiation and recovery progress is summarized in **Supplementary Table 1** for intuitive review. The daily dynamic changes of pancreatic enzymes, blood glucose and blood gas indicators during hospitalization are detailed in **Supplementary Table 2** for reference.

## 3 Discussion

This case clearly demonstrates that the PD-1 inhibitor toripalimab can simultaneously attack both the endocrine (islet β-cells) and exocrine (acinar cells) tissues of the pancreas, leading to the coexistence of acute pancreatitis and fulminant type 1 diabetes mellitus (FT1DM). Although this dual immune-mediated pancreatic injury phenotype remains extremely uncommon, cumulative clinical and epidemiological data published from 2023 to 2026 highlight its vital clinical relevance for frontline oncologists and endocrinologists.

Recent large-scale meta-analyses and multicenter real-world cohort studies have refined our understanding of the full clinical spectrum of ICI-related pancreatic injury. A 2024 meta-analysis pooling data from more than 48,000 ICI-treated patients reported the overall incidence of any pancreatic immune-related adverse event reached 3.6%, with 59.4% of affected patients developing grade 3 or higher toxicities (7). Symptomatic clinical pancreatitis only accounts for a small subset of these events, with pooled rates ranging 0.3%–0.93% across trial populations; meanwhile, asymptomatic isolated amylase or lipase elevation is far more prevalent, affecting roughly 2.6%–4.7% of all patients receiving PD-1/PD-L1 blockade, and these silent biochemical abnormalities carry potential risk of subsequent overt pancreatic inflammation (8). Incidence further differs by treatment regimen: PD-1 inhibitor monotherapy carries higher pancreatic toxicity risk than CTLA-4 single-agent treatment, while chemoimmunotherapy combinations—such as the toripalimab plus paclitaxel-cisplatin regimen used in our patient—correlate with elevated odds of early-onset dual pancreatic endocrine-exocrine damage, a trend observed across retrospective head and neck tumor cohorts (9).

Across all published ICI pancreatic irAE presentations, two distinct injury patterns have been consistently categorized: isolated exocrine pancreatitis, isolated fulminant insulin-deficient diabetes, and the rare overlapping dual phenotype documented here. Recent narrative reviews and pharmacovigilance analyses confirm that concurrent pancreatitis and FT1DM represents the most severe end of this clinical spectrum, with disproportionately high rates of ICU admission and life-threatening diabetic ketoacidosis compared with single-organ pancreatic injury. Tumor subtype acts as an unrecognized modifying risk factor: most prior dual-injury case reports focused on renal cell carcinoma or melanoma patients, while our report fills a gap for locally advanced head and neck squamous cell carcinoma treated with first-cycle chemoimmunotherapy, a population rarely captured in existing pooled analyses.

Current consensus recommendations published in 2025 multidisciplinary guidelines separate diagnostic and management pathways for isolated ICI pancreatitis versus ICI-induced FT1DM, yet unified guidance for overlapping dual pancreatic injury remains scarce (10). Standard NCCN irAE protocols recommend glucocorticoid intervention for grade ≥2 pancreatitis, while endocrine-specific consensus statements emphasize steroid avoidance where possible for FT1DM due to their ketogenic and insulin-resistance effects; this therapeutic conflict creates clinical uncertainty for patients presenting with both complications simultaneously. The following sections analyze our patient’s unique clinical profile, mechanistic hypotheses derived from existing translational work, and a stratified treatment framework balancing guideline recommendation against the metabolic vulnerabilities specific to concurrent pancreatitis and FT1DM.

It is worth emphasizing that ICI-mediated pancreatic immune injury presents two distinct clinical and radiological phenotypes: acute pancreatitis-like interstitial injury and autoimmune pancreatitis (AIP)-like inflammatory lesions, and differential identification of these two subtypes is critical for targeted clinical management. In our patient’s non-contrast abdominal CT scan, inflammatory changes were confined to focal swelling of the pancreatic body and tail accompanied by patchy peripancreatic fat exudation. There was no diffuse “sausage-like” enlargement of the entire pancreas, no hypodense peripheral capsule ring, and no segmental stenosis or irregular dilation of the pancreatic duct—imaging manifestations that are pathognomonic for type 1 autoimmune pancreatitis. In combination with the explosive onset of symptoms, marked elevation of serum amylase exceeding three times the upper limit of normal, and concurrent severe diabetic ketoacidosis, we completely excluded an AIP-like irAE phenotype and confirmed a diagnosis of immune-related acute interstitial edematous pancreatitis.

We also compared the diagnostic performance of amylase and lipase in this case. Lipase is widely accepted as a more pancreas-specific marker, as its elevation is rarely triggered by non-pancreatic sources such as salivary gland injury or small bowel disease, which are common causes of isolated amylase elevation. Lipase also remains elevated for a longer time after pancreatic inflammation sets in. In our patient, lipase was only slightly above the reference range, whereas amylase rose to more than three times the upper normal limit. The simultaneous rise of both enzymes, paired with characteristic pancreatic changes on non-contrast CT, gave us solid grounds to confirm immune-related acute pancreatitis. During daily rounds, we tracked serial amylase levels as our primary monitoring marker because amylase responds faster to fasting and somatostatin treatment, letting us adjust supportive care promptly based on real-time inflammatory changes.

ICIs are a major trigger for FT1DM. The core feature of FT1DM is the complete destruction of islet β-cells within an extremely short period. By relieving the inhibitory state of T cells, ICIs cause them to erroneously recognize islet β-cells as “non-self” antigens, thereby initiating an immune attack (11). Our patient’s normal HbA1c, negative islet autoantibodies, and absent C-peptide fully met the diagnostic criteria for FT1DM (5). Notably, Fang et al. published the only prior toripalimab-associated concurrent pancreatitis and FT1DM case in 2023 (12). Their patient suffered from clear cell renal cell carcinoma and developed dual pancreatic injury after four cycles of single-agent toripalimab monotherapy administered every two weeks over 11 weeks of treatment. While that report confirmed toripalimab could induce combined pancreatic endocrine and exocrine damage, our case carries several unique, previously unreported clinical observations that add critical new information to the existing literature. First, our patient had locally advanced base-of-tongue squamous cell carcinoma (cT2N2M0, Stage IVA) and experienced dual pancreatic injury just seven days following his very first cycle of toripalimab plus paclitaxel-cisplatin combination chemoimmunotherapy. This observation highlights that chemoimmunotherapy regimens may drive ultra-early, rapid-onset pancreatic toxicity, a clinical scenario absent from the earlier single-agent toripalimab case. Second, symptom presentation differed markedly: the previously published patient reported severe abdominal pain as his primary complaint, whereas our patient’s predominant manifestations were intractable vomiting, lethargy and impaired consciousness, with only mild epigastric tenderness. This atypical presentation creates a high risk that clinicians will overlook concurrent pancreatitis and solely diagnose uncomplicated diabetic ketoacidosis, delivering a key clinical warning for providers managing head and neck tumor patients on immunotherapy. Third, our patient achieved faster clinical recovery under standardized steroid-free supportive care. Pancreatic enzyme levels and ketosis resolved within 11 days in our cohort, whereas the prior toripalimab case required two full weeks for pancreatic enzymes to normalize. This comparison demonstrates that early, standardized intervention including nil per os management, somatostatin-mediated suppression of pancreatic secretion, and intensive insulin pump glycemic control can accelerate recovery for patients with mild interstitial edematous pancreatitis complicated by FT1DM.

To date, only two published cases worldwide describe concurrent toripalimab-induced acute pancreatitis and fulminant type 1 diabetes mellitus. Our report represents the first documented occurrence in a patient with head and neck squamous cell carcinoma receiving combined chemoimmunotherapy, filling an unmet gap in safety data for this common tumor subgroup treated with PD-1 inhibitor combination regimens. The divergent clinical profiles between the two toripalimab-associated cases also suggest that primary tumor type and concurrent chemotherapy exposure may modify the timing, symptom pattern and clinical trajectory of this rare immune-related adverse event, requiring heightened tailored monitoring across distinct patient populations.

The underlying mechanism may be related to the role of the PD-1/PD-L1 pathway in maintaining peripheral immune tolerance. The pathological process is intuitively illustrated in **Figure 2**, a mechanism diagram of pancreatic dual damage after PD-1 inhibitor treatment. Blockade of the PD-1 pathway activates T cells, which cause combined injury to pancreatic acinar cells and islet β-cells, resulting in acute pancreatitis and fulminant type 1 diabetes mellitus. Under physiological conditions, PD-L1 is expressed on islet β-cells and binds to PD-1 on T cells to inhibit the activation of autoreactive T cells, thus protecting the islets from immune attacks (12). When PD-1 inhibitors are administered, this “brake” mechanism is released, leading to the infiltration of activated CD8^+^ T cells into the pancreas, which simultaneously destroy acinar cells (causing pancreatitis) and β-cells (causing diabetes) (11). Animal model studies provide direct evidence for this: PD-1 gene-knockout non-obese diabetic (NOD) mice develop autoimmune diabetes at an accelerated rate (13).

**Figure 2:**
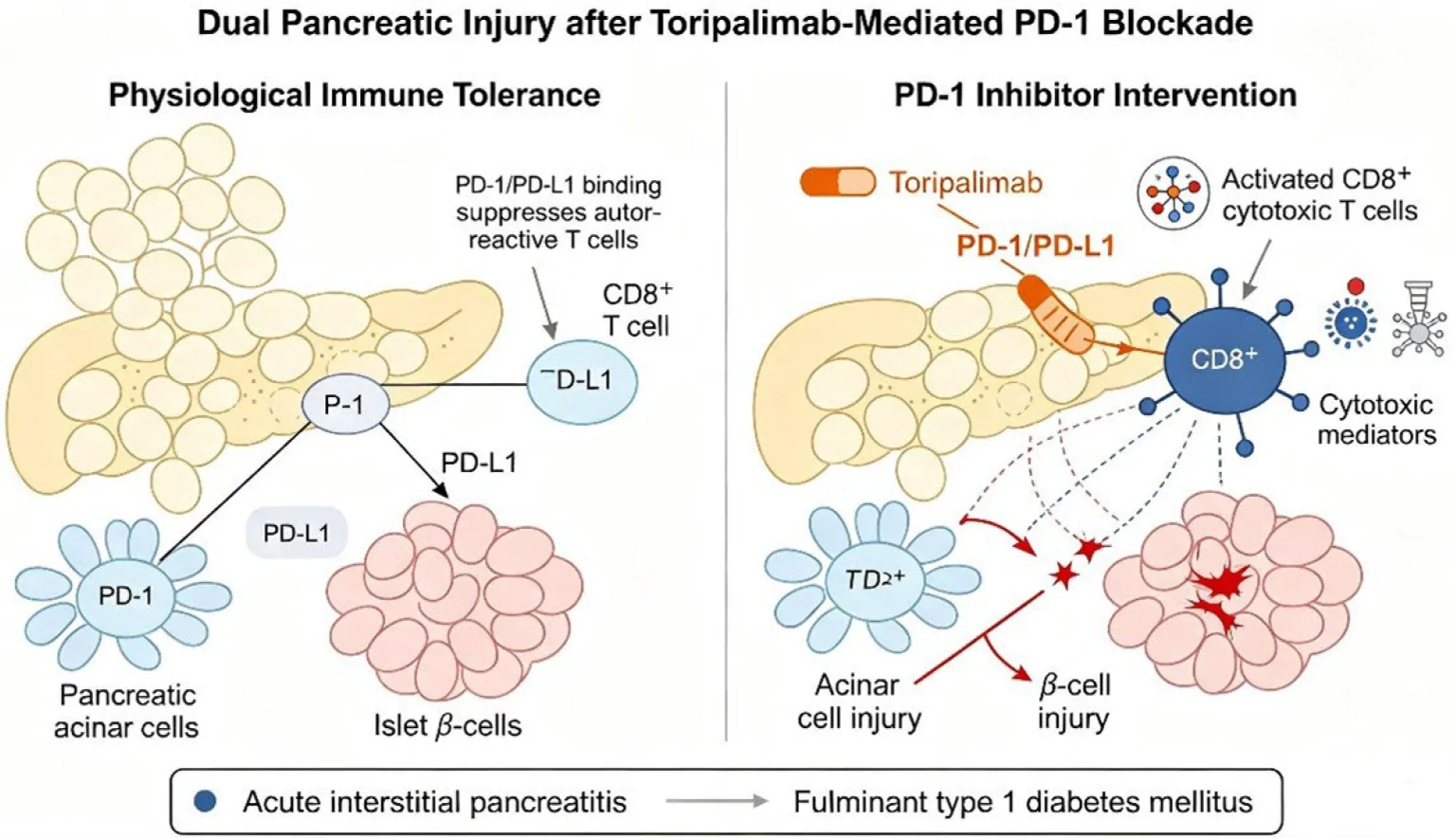
Color-coded, fully labeled hypothetical schematic showing dual pancreatic endocrine and exocrine injury after toripalimab-mediated PD-1 blockade. This image is a literature-derived mechanistic hypothesis; no pancreatic histopathology from this patient is available to validate this pathway directly. Left panel (physiological immune tolerance state): Color-coded annotated compartments mark pancreatic acinar cells (light beige), islet β-cells (pale pink), and CD8^+^ T lymphocytes (light blue). Explicit text labels connect PD-L1 expressed on pancreatic cell surfaces and PD-1 receptors on T cells, illustrating this binding axis suppresses autoreactive T cell activation to safeguard pancreatic tissue. Right panel (PD-1 inhibitor intervention state): Toripalimab (labeled orange blocking antibody) disrupts PD-1/PD-L1 binding. Activated CD8^+^ T cells (dark blue, marked with cytotoxicity markers) infiltrate pancreatic tissue, separately damaging acinar exocrine cells and endocrine β-cells, which sequentially triggers acute interstitial pancreatitis and irreversible fulminant type 1 diabetes mellitus. All core cellular subsets and checkpoint molecules carry distinct text callouts for unambiguous identification.

Regarding treatment strategies, this dual pancreatic irAE poses unique therapeutic dilemmas that require balanced consideration of guideline recommendations and patient-specific metabolic status. Current NCCN clinical practice guidelines recommend glucocorticoid intervention for Grade ≥2 immune-related pancreatitis (6). However, systemic corticosteroids carry pronounced diabetogenic effects that create substantial risks in patients complicated with concurrent fulminant type 1 diabetes mellitus (FT1DM), which explains our decision to withhold steroid therapy in this particular patient. Three critical clinical factors justified avoiding immediate glucocorticoids on admission. First, the patient presented with severe decompensated diabetic ketoacidosis, with an arterial pH as low as 7.28. Glucocorticoids stimulate hepatic gluconeogenesis and accelerate ketone body production, which would prolong acidosis correction and exacerbate metabolic decompensation. Second, abdominal CT only demonstrated mild interstitial edematous pancreatitis; there was no evidence of pancreatic parenchymal necrosis, widespread peripancreatic infection, or multiple organ dysfunction, consistent with low-to-moderate severity immune pancreatitis. Third, laboratory testing confirmed complete irreversible destruction of pancreatic β-cells, with undetectable fasting C-peptide. Exogenous steroids would induce severe peripheral insulin resistance, drastically elevate insulin requirements, and trigger unstable, difficult-to-control glycemic swings during acute hospitalization.

To avoid overgeneralized treatment claims derived only from our single case, we propose a stratified framework for steroid administration based on published guidelines and the two available toripalimab-associated combined pancreatitis-FT1DM case reports. For patients presenting with mild-to-moderate interstitial pancreatitis without necrosis or secondary infection, as seen in our case and the previously reported renal cell carcinoma patient, steroid-free supportive care represents a reasonable first-line approach. This regimen consisting of absolute fasting, targeted somatostatin infusion to suppress pancreatic exocrine secretion, early aggressive fluid resuscitation, and continuous subcutaneous insulin pump therapy safely resolved both pancreatic inflammation and ketoacidosis in both documented toripalimab cases. Conversely, if imaging identifies extensive pancreatic necrosis, secondary intra-abdominal infection, or progressive respiratory/renal organ failure, low-dose glucocorticoids can be introduced cautiously. In such scenarios, clinicians must implement hourly blood glucose surveillance and upward titration of insulin to offset steroid-driven hyperglycemia and ketogenesis. We emphasize that our favorable steroid-free recovery outcome cannot be universally extrapolated to all patients with this rare dual pancreatic injury; larger, multicenter cohorts are required to validate the safety and efficacy of this tiered management algorithm.

In conclusion, this case reiterates that clinicians must maintain a high index of suspicion for any patient undergoing ICI therapy. Once symptoms such as abdominal pain, nausea, vomiting, unexplained hyperglycemia, or ketosis occur, a comprehensive “pancreatic function panel” should be performed immediately, including serum amylase, lipase, blood glucose, blood ketones, HbA1c, and abdominal imaging. A normal HbA1c level is key to differentiating FT1DM from routine hyperglycemia. Early recognition, prompt drug discontinuation, and individualized supportive care form the cornerstone of improving the prognosis for such patients.

This study has several inherent limitations that should be acknowledged. First, due to the patient’s critical condition upon ICU admission complicated by severe decompensated diabetic ketoacidosis and unstable hemodynamics, contrast-enhanced abdominal CT, MRCP, and invasive examinations including EUS and ERCP were not clinically feasible, which restricted further radiological differentiation of pancreatic lesions. Nevertheless, we systematically screened for common alternative etiologies of pancreatitis by reviewing the patient’s medical history, liver and biliary biochemistry, and serum lipid levels, effectively excluding gallstone pancreatitis, alcoholic pancreatitis, and hypertriglyceridemia-induced pancreatitis in accordance with AGA diagnostic criteria for ICI-related pancreatic toxicity. Second, pancreatic histopathological biopsy was not performed, so the proposed immune injury pathway shown in **Figure 2** is a theoretical hypothesis synthesized from existing preclinical and clinical literature rather than direct pathological evidence obtained from this patient. Third, this is a single-center single-case observation, and prospective large-scale cohort data are still needed to validate the stratified steroid-free supportive management strategy we applied for combined pancreatic endocrine-exocrine irAEs.

## 4 Conclusion

Toripalimab can induce a rare but fatal dual impairment of both the endocrine and exocrine functions of the pancreas, manifesting as acute pancreatitis concurrent with fulminant type 1 diabetes mellitus (FT1DM). This complication is characterized by an abrupt onset, rapid progression, and severe clinical consequences, making it highly susceptible to being missed in clinical practice. In clinical settings, enhanced monitoring should be implemented for patients receiving PD-1/PD-L1 inhibitor therapy. Once suspected, emergency evaluation of pancreatic function is warranted.

Regarding treatment, permanent discontinuation of the offending agent and prioritization of individualized regimens centered on supportive care (fluid resuscitation, intensive insulin therapy, and inhibition of pancreatic enzymes) may serve as a safe first-line strategy for patients with mild interstitial pancreatitis combined with FT1DM, rather than indiscriminate early administration of glucocorticoids; stratified steroid use with tight glycemic monitoring should be reserved for patients complicated by pancreatic necrosis or severe secondary infection. Further large-scale clinical observations are needed to confirm the broad applicability of this supportive care-centered approach.

## Data Availability

The original contributions presented in the study are included in the article/**Supplementary Material**. Further inquiries can be directed to the corresponding author.
