# Supplementary material for: Immune checkpoint inhibitor–related pancreatitis with fulminant type 1 diabetes mellitus: a case report
Source: Front Immunol. 2026 Sep 15;17:1906731. doi: 10.3389/fimmu.2026.1906731 (PMC13619575; doi:10.3389/fimmu.2026.1906731)
Supplement: Supplementary file 1 [file Table1.docx]

Supplementary **Table S1**: Clinical Event Timeline of the Patient

| **Time Point** | **Key Clinical Events** |
| --- | --- |
| Jan 12, 2026 | First cycle antitumor therapy: toripalimab 240 mg ivgtt d1 + TP chemotherapy (paclitaxel + cisplatin); no acute infusion adverse reactions. |
| Jan 17, 2026 (Day 5 post-treatment) | Gradual onset of dizziness, fatigue, poor appetite. |
| Jan 19, 2026 (Day 7 post-treatment, early morning) | Sudden recurrent nausea/vomiting of gastric contents, dry mouth, polydipsia, lethargy and slow response; transferred to local hospital for emergency blood tests, revealed hyperglycemia, urine ketone (+++), metabolic acidosis; transferred to our ICU immediately. |
| Jan 19, 2026 (On ICU admission) | Complete emergency laboratory panel: random blood glucose 17.40 mmol/L, blood ketones 9.52 mmol/L, arterial pH 7.28, serum amylase 370 U/L; upper abdominal non-contrast CT performed, showing swelling of pancreatic body/tail with peripancreatic exudation. |
| Jan 19, 2026 (After admission evaluation) | Combined diagnosis confirmed: Grade 3 toripalimab-related acute pancreatitis + toripalimab-associated fulminant type 1 diabetes mellitus; toripalimab and subsequent chemotherapy permanently discontinued. Initiate full supportive management: strict fasting, gastrointestinal decompression, somatostatin continuous infusion, pantoprazole acid suppression, prophylactic piperacillin-tazobactam, continuous subcutaneous insulin pump. |
| Jan 20, 2026 (Treatment Day 2) | Nausea and vomiting fully relieved, mental status markedly improved. |
| Jan 21, 2026 (Treatment Day 3) | Blood ketones turned negative, arterial blood gas pH returned to normal, diabetic ketoacidosis resolved. |
| Jan 23, 2026 (Treatment Day 5) | Serum amylase decreased to 128 U/L, significant alleviation of pancreatic inflammation. |
| Jan 29, 2026 (Treatment Day 11) | Stable general condition, pancreatic enzymes and blood glucose maintained within normal range; insulin pump switched to multiple daily insulin injection scheme (glargine at bedtime + aspartate before three meals); patient discharged. |
