# Supplementary material for: Immune checkpoint inhibitor–related pancreatitis with fulminant type 1 diabetes mellitus: a case report
Source: Front Immunol. 2026 Sep 15;17:1906731. doi: 10.3389/fimmu.2026.1906731 (PMC13619575; doi:10.3389/fimmu.2026.1906731)
Supplement: Supplementary file 2 [file Table2.docx]

Supplementary Table S2: Serial Laboratory Dynamic Changes During Hospitalization

| **Hospital Day** | **Date** | **Serum amylase (U/L, ref <100)** | **Lipase (U/L, ref <60)** | **Random blood glucose (mmol/L)** | **Blood ketones (mmol/L)** | **Arterial pH** | **HCO₃⁻ (mmol/L)** | **Fasting C-peptide (ng/mL, ref 1.1–4.4)** | **HbA1c (%)** |
| --- | --- | --- | --- | --- | --- | --- | --- | --- | --- |
| Admission Day 1 | 2026-01-19 | 370 | 68 | 17.40 | 9.52 | 7.28 | 14.2 | <0.05 | 5.4 |
| Treatment Day 2 | 2026-01-20 | 246 | 62 | 11.30 | 3.15 | 7.34 | 19.6 | — | — |
| Treatment Day 3 | 2026-01-21 | 161 | 54 | 8.70 | 0.08 | 7.39 | 23.8 | — | — |
| Treatment Day 5 | 2026-01-23 | 128 | 47 | 7.50 | <0.01 | 7.41 | 24.5 | — | — |
| Treatment Day 8 | 2026-01-26 | 97 | 39 | 7.10 | <0.01 | 7.42 | 25.1 | — | — |
| Discharge Day 11 | 2026-01-29 | 83 | 32 | 6.60 (fasting) / 9.20 (postprandial) | <0.01 | 7.42 | 25.3 | — | — |
